# Development of biochar/HDPE composites and characterization of the effects of carbon loadings on the electromagnetic shielding properties

**DOI:** 10.1016/j.heliyon.2024.e24424

**Published:** 2024-01-11

**Authors:** Amanu Asmare Fenta, Addisu Negash Ali

**Affiliations:** Faculty of Mechanical and Industrial Engineering, Bahir Dar Institute of Technology, Bahir Dar University, P.O.Box26, Bahir Dar, Ethiopia

**Keywords:** Biochar/HDPE composite, Conductivity, Electromagnetic wave, Shielding effectiveness properties

## Abstract

The aim of this research is to develop high carbon-yielding biochar from pinewood, coffee husk, sugarcane bagasse, and maize cob and to characterize the biochar/HDPE composites for electromagnetic (EM) shielding application. During the biochar/HDPE composites fabrication, slow pyrolysis and compression molding manufacturing were used. The enhanced properties characterizations were conducted by using thermogravimetric analysis (TGA), scanning electron microscopy (SEM), differential thermal analysis (DTA), Fourier transform spectrometry (FTIR), Brunauer-Emmet-Teller (BET) analysis, digital multi-meter, and proximity analysis. The results of biochar pyrolysis showed the maximum carbon yield of 74.6 %, 68.9 %, 68.4 %, and 40 % for pine wood, maize cob, sugarcane bagasse, and coffee husk respectively. The BET analysis showed the maximum specific surface area (734.5 m^2^/g), pore volume (0.2364 cm^3^/g), and pore radius (9.897 Å) from the pine wood biochar. The biochar loading analysis results showed that the 30 % and 40 % pine wood biochar significantly enhanced the electrical conductivity, thermal conductivity, thermal stability, crystallinity, and EM shielding effectiveness (SE) of the biochar/HDPE composites. In particular, the biochar/HDPE composite with 30 wt% pine wood biochar showed the highest thermal conductivity of 2.219 W/mK, and the 40 wt% pine wood biochar/HDPE composite achieved the highest electrical conductivity of 4.67 × 10^−7^ S/cm and EM SE of 44.03 dB at 2.1 GHz.

## 1 Introduction

Electromagnetic (EM) wave is an energy emitted from different sources. The sun is the primary source of EM wave and the other common EM wave sources are electricity, appliances, cell phones, satellites, telecommunication centers, power lines, and other electronic devices [1]. EM waves can interfere with other devices and cause the reduction of their efficiency leading to malfunctioning and loss of stored data [2,3]**.** Currently, EM pollution needs urgent solution due to its harmful effect on the electronic equipment, environmental ecosystem, and human health [4]. EM shielding prevents the penetration of wave or radiation into electronics devices and human body. It is the method of controlling EM wave by blocking the radiation field using barriers composed of conductive materials which is commonly known as EM shielding [5]. During the EM shielding, the materials morphological structure plays a great role [6]. The EM wave power loss due to the shielding material is called as the shielding effectiveness (SE) and expressed by decibel (dB). The main methods of EM shielding are reflection, absorption, and multiple reflections [7]. During the absorption EM shielding, the materials absorb and dissipate the EM waves in the form of current and heat due to the interaction with electric or magnetic dipole of the material [8]. The second mechanism is the reflection of waves which can occurs due to the interaction between EM waves and charge carriers in a highly conductive structures [9,10].

Different researches are ongoing on the shielding solutions to alleviate the challenges of EM waves [11]**.** Over the past years, researchers used metals, which can provide high electrical conductivity, and good mechanical properties for reflective EM shielding application [12]**.** Most commonly, metals such as copper, aluminum, and nickel were used as reflective EM shield materials [13]**.** The limitations of these metals in the EM shielding application were considered to develop metals hybrid with polymers, fabrics, and aerogels [14]. However, metals have drawbacks like high density, corrosion susceptibility, limited availability, high cost, and processing difficulties [15]**.** Increasing the EM absorption of materials can increase the shielding effectiveness (SE) of EM waves [16]. Recently, researchers have started introducing shielding effective materials like carbon, polymers, graphene, and composites [15]. Due to their outstanding electrical conductivity, high aspect ratio, and controllable geometry, one-dimensional materials such as CNTs, and carbon nano-fibers have been widely used for high-performance EM shielding application [3]. Especially, carbon-based filler with polymer matrix are the most effective electromagnetic shielding material because of their high absorption, high reflection, low cost, and abundant availability [17].

Recently, biochar has attracted the scientific community not only for soil amendment and fuel extraction applications [18] but also as a carbonaceous source for high-value-added applications. Biochar is known to have porous nano/micro-structures [19], abundant functional groups [20], various inorganic nutrients [21], and high carbon stability [19]. Wood, agricultural residue, poultry residue, forest residue, agro-industry wastes, and animal wastes are among the biomass feed stocks that can be used to produce high carbon yielding biochar. Biochar can be fabricated as solid using pyrolysis and gasification of biomass, or as a slurry by hydrothermal carbonization at high pressure [22]. In the previous works, biochar was used as an excellent choice for strengthening wide ranges of thermoset, and thermoplastic polymeric matrix materials [23]. In this research, the biochar is prepared from pine wood, coffee husk, sugarcane bagasse and corn cobs integrated with the recycled high density polyethylene (HDPE) [24,25]. Coffee is one of the world's populous plant and approximately 7 million tons of coffee is produced per year [26] globally**.** Coffee husk consists of holocellulose (44.9 %), lignin (28.3 %), other extractives (26.2 %), and ashes (0.6 %) [27]. Woody biomass is a primary source of biochar production worldwide. Hemicellulose, cellulose, lignin, and minor amounts of organic extractives such as lipids, phytosterols, and phenolic as well as inorganic components such as nitrogen, phosphorus, sulfur, silicon, earth metals, and different trace minerals are the constitutes of the woody biomass. The production of sugar from sugarcane has the main byproduct of bagasse. The production of biochar from these and the like agricultural and agro-industry wastes should be the prior choice for different value-adding applications [28,29]. Maize cob residues contain the main constituent of lignocellulose materials (76.7 %); cellulose and lignin (14.7 %), extractive contents (7 %), and ash around (1.6 %) [27]. The use of maize cob residues biochar for EM shielding application is much cheaper, cost-effective, and environmentally friendly.

Pyrolysis is the most prevalent and the easiest biomass conversion technology to produce biochar with high carbon yield and characteristics [30]. In the pyrolysis process, biomass is thermo-chemically transformed into bio-oil, charcoal, and gas in an oxygen-free inert atmosphere using either conventional or microwave heating systems at various temperatures and pyrolysis periods. Pyrolysis is mostly used to develop liquid bio-oil, and charcoal as a byproduct. During pyrolysis, hemicellulose breakdowns at a temperature of 220–315 °C, cellulose decomposes at 315–400 °C, and lignin degrades at the highest temperature of around 900 °C [31]. Based on the heating rate and residence time, pyrolysis can be divided into two types; slow and fast [32]. Slow pyrolysis has a long residence time (from a few minutes to several days) and low temperature range (300–500 °C) [33]. Fast pyrolysis has been investigated extensively for the production of renewable liquid fuels that can be utilized as a substitute to petroleum or as a chemical feedstock [33]. For gas-phase products, dried biomass (less than 10 % by weight of moisture content) is heated at a faster heating rate (above 200 °C min^−1^) and shorter residence time (less than 10 s). Fast pyrolysis produces 60–70 % liquid bio-oil by weight, 15–25 % biochar by weight, and 10–20 % non-condensable gases by weight [34].

EM pollution is the fourth next to air, water and sound pollution. Currently, different EM shielding materials are under investigations. However, their cost, flexibility, accessibility, manufacturability, and environmental safety are still problems. Therefore, the key innovation of this research is the plan to use abundantly available sustainable biomass resources to develop effective EM shielding materials. For EM shielding application, materials should have the properties of good electrical conductivity, good thermal conductivity, and high porosity values. The main objective of this research is to fabricate, and characterize the biochar/HDPE composites at different biochar loadings for EM shielding application. In this work, high pyrolysis temperature biochars are developed from different biomasses with high thermal stability, high conductivity, and excellent EM shielding properties. The biochars are mixed with HDPE to develop solid composites with proper structural integrity. The fabrication of biochar/HDPE composites is applied by using the newly designed and developed pyrolysis setups and subsequent compression molding. Experimental characterizations by using TGA, SEM, DTA, FTIR, BET, and proximity analysis will be applied to investigate the EM of each biochar/HDPE composites.

## 2 Materials and methods

### 2.1 Materials

The raw materials used for the production of EM shielding composites were pine wood, coffee husk, sugarcane bagasse, maize cobs, and recycled HDPE. The coffee husk (CH) was collected from a local coffee milling industry in Zegie, Bahir Dar, Ethiopia. The second biomass used was pine wood collected from the local furniture enterprises in Bahir Dar. The third biomass was maize cob collected from Motta, East Gojjam zone, Amhara region. The fourth biomass was sugarcane bagasse collected from sugarcane juice processing laboratory in Bahir Dar University. Finally, all the collected biomasses were sun dried for one day to reduce moisture content for pyrolysis application. The recycled high density polyethylene (HDPE) plastic was used as the matrix of the composites. The HDPE plastic waste was collected from Amhara Pipe Factory in Bahir Dar with the properties listed in Table 1. The pine wood, coffee husk, sugarcane bagasse, and maize cob biochars were grounded into a 150 μm average size and sieved to obtain uniform size powders. For EM shielding applications, the size of the filler should be as small as possible.

**Table 1:** Properties of the recycled HDPE [35].

Property	Value
**Melting point**	160–240 °C
**Density**	0.734–0.97 g/cm^3^
**Tensile strength**	21.9 MPa
**Electrical resistivity**	10^15^-10^18^ Ωcm

### 2.2 Design and manufacturing of the pyrolysis setup

**Fig. 1:**
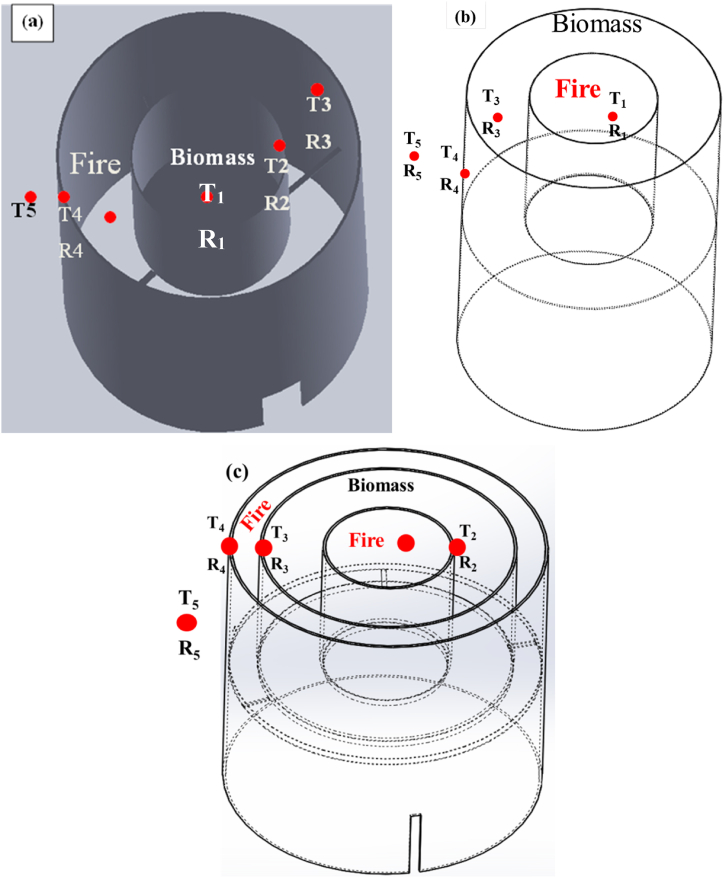
Pyrolysis experimental setup design by considering (a) inner heat supply, (b) outer heat supply, and (c) heat supply from both sides.

The pyrolysis setup was designed by considering three conditions as shown in Fig. 1. The best biochar pyrolysis production setup was selected from three designed setups based on the theoretical values of the design temperature and uniformity of heat distribution throughout the pyrolysis process. During the design of the pyrolysis setup, the physical and thermal properties of the biomasses were considered to select the setup construction materials as shown in Table 2.

**Table 2:** Properties of materials for pyrolysis setup design considerations.

Materials Properties	Fire Wood	Coffee husk	Pine wood	Maize corn cob	Sugarcane bagasse	Mild steel
**Density (g/cm**^**3**^**)**	0.495	0.25	0.5	0.227	0.096	7.89
**Thermal conductivity k (W/mK)**	0.30	0.19	0.12	0.162	0.08	50
**Specific heat value (KJ/Kg K)**	1.017	1.62	–	0.6085	–	–
**Heating value (MJ/Kg)**	17.5	18.34	–	–	–	–
**References**	[36]	[37]	[38]	[39]	[40]	[41]

During the biochar production, the forced convection coefficient of the flame was assumed to be 60 W/m^2^K which can be varied in the range of 10–500 W/m^2^K (standard values). The forced convection assumption was considered due to the presence of a 1.4 m/s average wind speed measured at Bahir Dar University, Bahir Dar, Ethiopia which can affect the continuous heat supply of biomass firing [42]. During the pyrolysis design, the isothermal internal heat was assumed which is equivalent to the heating value of the firewood supplied for the given pyrolysis periods. Furthermore, during combustion heat was assumed to be transferred radially in the horizontal direction. Therefore, for the first pyrolysis setup as shown in Fig. 1 (a), the amount of the required firewood was calculated to be around 17.49 kg which is sufficient enough to change the biomass into biochar with the temperature ranges of 515.5–621.8 °C inside the combustion chamber. The fire wood temperature ranges were determined based on the peak fire flame temperature measurements [42]. The isothermal internal burning can give heat equal to the heating value of the wood supplied times the amount of wood required for the given pyrolysis periods. With similar combustion conditions, the second pyrolysis type as shown in Fig. 1 (b) was designed with 24.49 kg of firewood to change the biomass into biochar. For the case of heat supply from both sides, the 3rd type of pyrolysis setup as shown in Fig. 1 (c) was designed. In the 3rd case, the area of the internal and outer heat suppliers should be equal for equal heating from both sides. Hence, internal and external areas are taken to be equal and a total of 24.49 kg of firewood is required to change the biomass effectively into full biochar. The 3rd pyrolysis setup has uniform heat supply and high temperature heating capacity. Therefore, in this research the 3rd pyrolysis setup was used to produce the coffee husk, pine wood, sugarcane bagasse, and maize cob biochars with effective pyrolysis temperature of 641.57 °C. Based on the combustion requirements, mild steel with tensile strength of 250 MPa, melting point of 1450 °C, thermal conductivity of 50 W/mK, convection coefficient of 25.32 W/m^2^K, and density of 7.89 g/cm^3^ was been selected for pyrolysis setups construction.

### 2.3 Physical and chemical properties analysis of biochar

The physical and chemical properties of the coffee husk, pine wood, sugarcane bagasse, and maize cob biochars were characterized to understand the effect of pyrolysis time and biomass sources on the char quality, carbon content, surface area, pore diameter, and pore volume to select the best biochar filler for biochar/HDPE composites for EM shielding application. The proximate analysis was conducted by using muffle furnace by following the standard rules of measuring the fixed carbon. First, the moisture content was measured by heating the biochars at 105 °C for 30 min using oven dryer (DHG-9053A- 2018). Then, the mass left after moisture removal was measured by electronic mass balance (JA2603 N, SN: Q01201701030). The same masses were put into the muffle furnace again and the temperature was set to 950 °C and dried for 8 min. After recoding the mass, it was put into the muffle furnace and the temperature of the furnace was set to 750 °C to measure volatile matter (VM) and ash contents. The volatile matter and ash contents were determined using the ASTM D3172-13 methods [43]. Based on ASTM D3172-13 methods, the VM content was determined as a weight loss after heating the moisture-free biochar in a covered crucible at 950 ± 20 °C and holding it for 8 min. Furthermore, the specific surface area and pore size of each biochar was measured by using Brunauer-Emmet-Teller (BET) equipment. Specific surface area was determined based on the absorption of N_2_ molecules in the surface of the microstructures of the biochar. The surface area was determined using the BET method by considering 0.05 to 0.45 relative pressures (P/P_o_). The pore size distribution was determined using the Barret-Joyner-Haelender (BJH) method at a relative pressure (P/P_o_) of 0.99. Finally, the biochar with high carbon content, specific surface area, pore diameter, and pore volume was selected as filler materials for EM shielding application. Due to its high carbon content compared to coffee husk, maize corn, and sugarcane bagasse, the pine wood was selected for further analysis and characterization.

The elemental composition of pine wood biochar was determined by following the standard procedures of atomic spectrum analysis. A 0.5 g of pine wood biochar powder was digested and placed in a flask which aids for the removal of all organic components of the biochar. During the digestion, nitric acid, hydrogen per oxide, and chloric acid at mixing ratios of 2:1:0.5 respectively were used as solvent. The digestion was performed on the hot plate digester for 2 h and 40 min [44]. The chemical functional groups of the pine wood biochar/HDPE composites were investigated using FTIR (FT/IR-6600). The FTIR spectrum of the composites were measured and compared with the values in the literatures. Furthermore, the surface morphology and 2D structures of pore distributions of the composites were examined by using JCM-6000 scanning electron microscope (SEM). Furthermore, the pine wood biochar particles and HDPE plastic resin composites were manufactured at different weight percentages based on the rule of mixtures [27] as shown in Table 3. The mixing ratio of the composite is taken based on the literature which took 1–80 % of biochar composition to mix with the HDPE. For example, Zhang, Q. et al. [45], used 30, 40, 50, 60 and 70 wt percentage (wt%) of biochar to melt and extrude with HDPE. The pine wood biochar and HDPE were mixed manually and melt blended at a temperature of 160 °C. Finally, the blended composite was molded by using hot compression molding using a nominal pressure of 25 MPa. The target size of the sample composites were 150 mm × 130 mm with a thickness of 3 mm.

**Table 3:** Weight percentages and mass of the constituent materials.

Sample	**Name of sample**	HDPE (%)	**Pine wood biochar (%)**	**Mass of PW biochar(g)**	**Mass of HDPE (g)**	**Mass of composite (g)**
**Sample 1**	Neat HDPE	100	0	0	94.575	94.575
**Sample 2**	PW-HDPE	90	10	8.847	79.625	88.472
**Sample 3**	40B	60	40	28.501	42.752	71.253
**Sample 4**	30B	70	30	20.125	51.959	77.084
**Sample 5**	20B	80	20	16.58	69.064	82.914
**Sample 6**	10B	90	10	8.874	79.866	88.745

Two methods were carried out to measure the EM SE of the fabricated composites. The first method was free-space transmission method which uses two horn antennas placed at a distance of 0.4 m connected to antenna measuring software in order to determine the SE and the samples were placed in between antennas. This method doesn't show the full effective SE of the samples because of its little area coverage of the sample which is 150 × 130 mm^2^. In the free-space EM shielding test, the scanned frequency was difficult to identify because of the uncontrollable state of free-space. The second method was by using the vector network analyzer (ZVK-1127.8651) and the transmitted signal with and without sample was measured at 10 MHz to 40 GHz based on the amount of power absorbed or reflected from the sample. For the measurements of the EM SE, six samples were fabricated based on ASTM D4935 standard dimension of rectangular wave guide samples (22.9 × 10.2 × 3 mm^3^ x 3 mm) [46]. The thermal stability of the biochar/HDPE composites were characterized by using HCT-1 thermal balance, Beijing Henven Instrument. The percentage weight loss was analyzed and the degradation temperature of the neat HDPE and its composites were determined [47]. The thermal conductivity of the biochar/HDPE composite samples was analyzed using a G.U.N.T WL 374 thermal conductivity device. Furthermore, a fluke 87 true RMS digital multi-meter was used to measure electrical conductivity of the biochar/HDPE composites using a 20 mm diameter and 3 mm thickness samples. The density of the constituents and the biochar/HDPE composites were determined using 80 mm length and 12 mm width samples according to ASTM D-1037-06a standard [27]. The density of each sample was evaluated by using oven drying at 105 °C for 30 min and weighing the sample by using a high precision electronic weighing balance.

## 3 Results and discussion

### 3.1 Pyrolysis conditions and biochar constituents

**Fig. 2:**
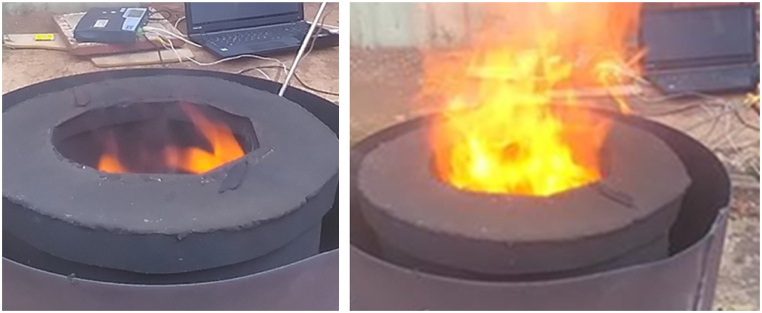
Pyrolysis process setups demonstrations.

**Fig. 3:**
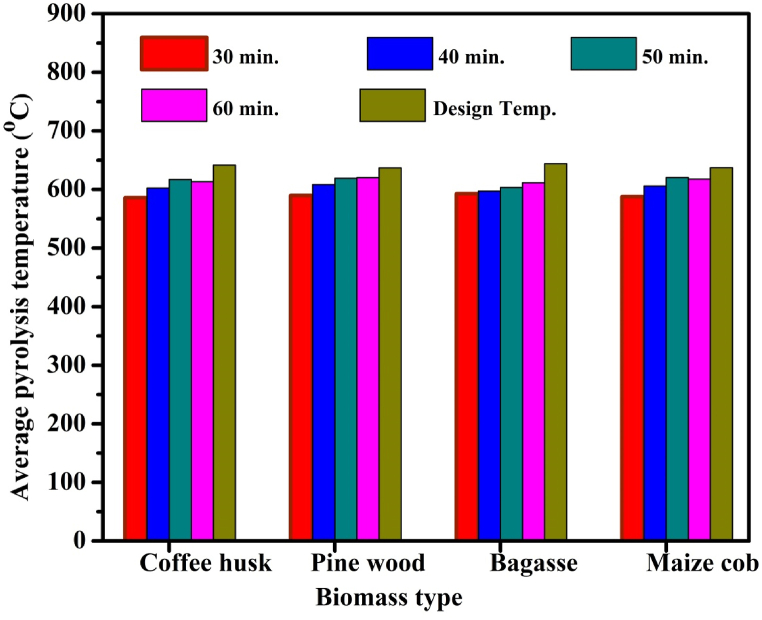
Average pyrolysis temperature measurement results of different biomasses at different pyrolysis periods.

To determine the optimum pyrolysis period, four different pyrolysis conditions (30, 40, 50 and 60 min) were investigated based on information from literature. For example, Nicholas, K. and S. Julius [48], used 30–60 min to pyrolyze coffee husk with batch bio-reactor under slow pyrolysis conditions in the temperature range of 350 °C-550 °C. The selection of the best pyrolysis time was done by considering the yield of biochar carbon. The maximum carbon content was obtained at 60 min of the pyrolysis periods and the average pyrolysis temperature was also determined, which was slightly lower than the calculated (design) pyrolysis temperature for all the four pyrolysis periods as shown in Fig. 3. The maximum carbon content for pine wood, maize cob, sugarcane bagasse, and coffee husk was 74.6 %, 68.9 %, 68.4 %, and 40 % respectively. The same carbon content of 74.6 % was obtained at both 50 and 60 min for pine wood biochar. The pyrolysis temperature measurement was conducted starting from the ambient temperature for 60 min by using thermocouple integrated with data logger installed at the internal surface of the biomass combustion compartment in the pyrolysis setup as shown in Fig. 2. The variation of temperature between the calculated values and the experimental results at 60 min of pyrolysis time was determined to be 3.36 %.

The proximate analysis was done to determine the volatile matter (VM), moisture content, ash content, and carbon content as shown in Table 4 at all the four pyrolysis periods for all the four biochar types. The results indicate that the carbon content has an increasing patterns corresponding to the pyrolysis time depending on the types of biomass sources. The maximum carbon content was obtained at 50 min of the pyrolysis time with no variations up to 60 min for the pine wood biochar (74.6 %) followed by the maize cobs biochar (68.87 %) at 60 min. As stated in the methodology section, the contents of VM and ash contents were determined using the ASTM D3172–13 methods [43]. As depicted in Table 4, the wastes such as VM, and ash content have been reduced at a higher pyrolysis time for all biochar types. Moreover, the VM, and ash content have been reduced significantly at 60 min of the pyrolysis time to 17.1 % and 2.6 % respectively for both pine wood and maize corn biochars.

**Table 4:** Basic parameters and composition proportions of CH, SB, MC, and PW biochars.

Parameters	Carbon content (%)			Moisture content (%)			Volatile matter (%)			Ash content (%)		
**Period (min.)**	40	50	60	40	50	60	40	50	60	40	50	60
**Coffee husk (CH)**	14.3	31.4	40.0	14.3	11.4	11.4	45.7	37.1	34.3	25.7	20	14.3
**Sugarcane bagasse (SB)**	54.3	65.7	68.4	8.57	5.7	8.6	31.4	25.7	20	5.7	2.9	2.9
**Maize cobs (MC)**	60.0	68.6	68.9	14.3	8.6	11.4	22.9	20	17.1	2.9	2.9	2.6
**Pine wood (PW)**	65.8	74.6	74.6	5.7	2.9	5.7	25.7	20	17.1	2.9	2.6	2.6

**Fig. 4:**
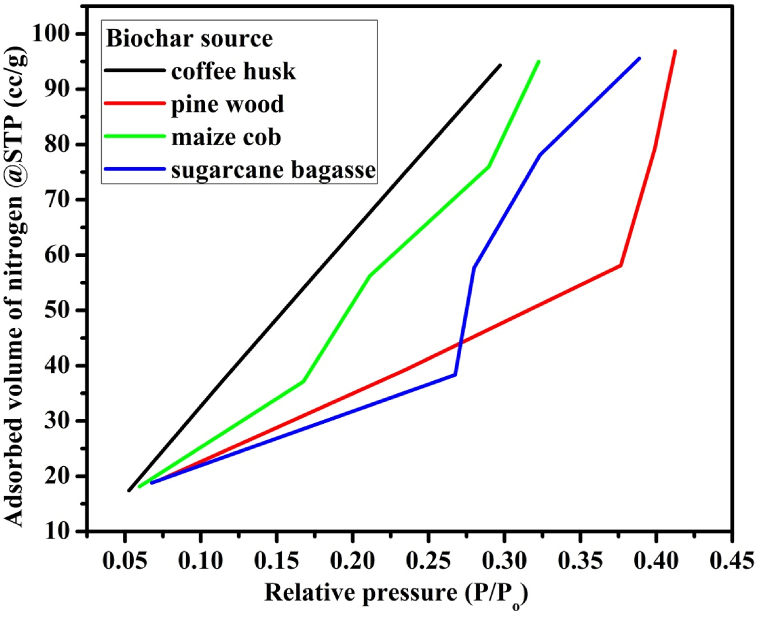
The BET curve for each biochar at various relative pressures.

The carbon content of the CH was determined to be much lower compared with the other biochar types, which was 14.3 %, and 40 % at 40 and 60 min of pyrolysis periods respectively. The lower carbon content of the CH biochar type might be related with the coffee varieties. The BET and BJH measurements were done to determine the absorbed nitrogen to specify the specific surface area and pore size of each biochar as shown in Fig. 4 and able 5 respectively. The biochar/HDPE composites’ important parameters such as pore size, pore volume, and specific surface area were determined by using quanta chrome NovaWin-Data Acquisition system as shown in Table 5. The high pore size, pore volume, and specific surface area have significant contributions on the EM shielding capacity of biochar/HDPE composites and on the micro/nano-structural integrity of biochar and HDPE. Nitrogen absorption measurements were made to characterize the PW, CH, SB, and MC biochar samples. As illustrated in Table 5, the maximum specific surface area (734.5 m^2^/g) was attained for the pine wood biochar compared to the other biochar types. Similarly, the highest pore volume (0.2364 cm^3^/g), and pore radius (9.897 Å) were recorded for the pine wood biochar type.

**Table 5:** Specific surface area, pore size, and pore volume of various biochar types.

Parameters	CH	PW	SB	MC
**Specific surface area (m**^**2**^**/g)**	535.5	734.5	598.6	612.3
**Pore volume (cm**^**3**^**/g)**	0.1364	0.2364	0.1676	0.2129
**Pore radius (Å)**	8.337	9.897	8.213	9.012

The multi-point specific surface area BET measurements gave 535.3, 734.5, 598.6, and 612.3 m^2^/g average specific surface area for coffee husk biochar (CHB), pine wood biochar (PWB), sugarcane bagasse biochar (SBB), and maize cobs biochar (MCB) respectively. As illustrated in Table 5, the nonlocal density functional theory (NLDFT) method was used for pore volume analysis, which gives 0.1364, 0.2364, 0.1676, and 0.2129 cm^3^/g pore volume for CHB, PWB, SBB, and MCB respectively. The pore radius size measurements also gave 8.337, 9.897, 8.213, and 9.012 Å for CHB, PWB, SBB, and MCB respectively. From the overall BET analysis, the pine wood biochar showed the highest specific surface area, pore volume and pore size. When we compared the BET analysis of pyrolyzed and non-pyrolyzed pine wood biochars, there were a 287.9 m^2^/g specific surface area, 0.1074 cm^3^/g pore volume, and a 1.32 Å pore radius differences. From previous literature, it was observed that as the pyrolysis temperature increased the specific surface area was also increased [49,50]. Furthermore, the heavy metals elemental analysis of PWB was applied using AA500 atomic absorption spectrophotometer flame and graphite analysis. The results indicated that no heavy metals (lead, cadmium and chromium) exist in the biochar samples.

**Fig. 5:**
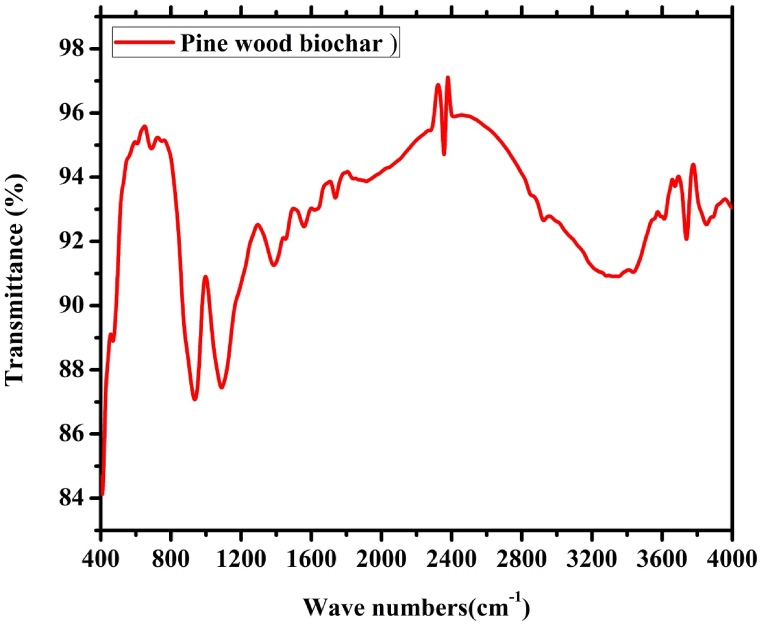
FTIR analysis results of the pine wood biochar pyrolized for 60 min.

**Fig. 6:**
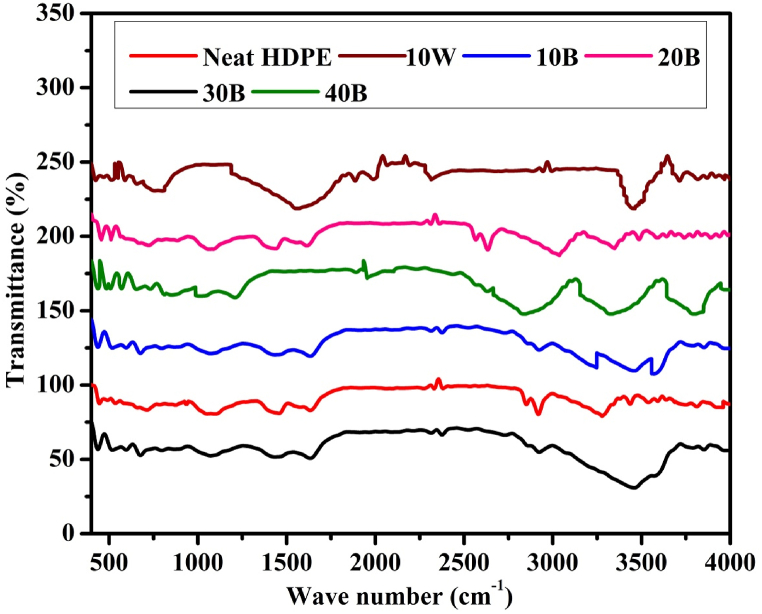
FTIR analysis of pine wood/HDPE composite and pine wood biochar/HDPE composites.

The functional groups of the PW biochars were investigated using Fourier Transform Spectroscopy (FTIR) as shown in Fig. 5, and Fig. 6. The FTIR spectra of pine wood biochar as shown in Fig. 5 showed a high transmittance values with a characteristic peaks in the wave number ranges of 600–800, and 2200-2600 cm^−1^, and also, small peaks were observed to occur at above 3200 cm^−1^ wave number. Within these wave number ranges, an aromatic, aliphatic, alcohols, phenols, and oxygen-containing functional groups were observed with a stretching state [51]. Specifically, the first peak was observed to occur at 735–770 cm^−1^ which consists of stretching aliphatic and phenols compounds [52]. It consists of stretching aliphatic phosphates, silicate ions, and vibrating cyclohexane ring [53]. And also, it consists of stretching non-bonded hydroxyl groups –OH, primary alcohol, secondary alcohols, and tertiary alcohols at the wave number ranges of 3645–3530 cm^−1^ [54,55].

The biochar/HDPE composites were analyzed by using FTIR spectroscopy to determine the reactions and change of functional groups in the blended composites during hot compression molding. As shown in Fig. 6, the pure HDPE has lower peaks compared with blended biochar/HDPE composites, and this can lead to conclude that most functional groups (peaks) of the composites are developed due to the addition of the pine wood biochar. From the FTIR analysis, almost no new functional groups or change of functional groups are observed due to the hot molding process [56]. However, the FTIR analysis of the un-pyrolyzed pine wood biochar/HDPE composites showed different peaks due to the presence of volatile matter, ash and other residues that need to be removed during pyrolysis [57]. As shown in Fig. 6, when the biochar content increased to 30 % and 40 %, the biochar/HDPE composites showed an increment of the number of small peaks. This confirms the blending reaction between biochar and HDPE is lower and no significant changes in the functional groups [58]. Specifically, functional groups including aliphatic and aromatic amines, nitriles, alkanes, alkenes, alkynes, alcohols, carboxylic acids, phenols, primary, secondary and tertiary alcohols and other residuals are available in the stretched, vibrated, and bended state in their position [59]. The existing state of the functional groups can contribute to electrical conductivity, thermal conductivity, and for the formation of crystallinity. The spectral wave bandwidth was decreased when the filler biochar is combined with HDPE matrix compared with the pure biochar [60].

As shown in Fig. 6, the 10 % pure pine wood/HDPE composite indicated the presence of significantly different spectra and peaks. The FTIR spectra analysis indicates range of characteristic peaks such as C–H 1,2-disubstitution at 735–770 cm^−1^, C–H mon substitution at 770–730 cm^−1^, aliphatic chloro compounds at 710–690 cm^−1^, C–Cl stretch at 800–700 cm^−1^, aliphatic bromo compounds, and C–Br stretch at 700–600 cm^−1^. It also consists of aliphatic phosphates (P–O–C stretch) from 1050 to 990 cm^−1^, silicate ion at 1100–900 cm^−1^, cyclohexane ring vibrations at 1055–1000/1005–925 cm^−1^, thiols (S–H stretch) at 2600–2550 cm^−1^, non-bonded hydroxyl group –OH stretch at 3645–3600 cm^−1^, primary alcohol –OH stretch at 3645–3630 cm^−1^, secondary alcohol –OH stretch at 3635–3620 cm^−1^, tertiary alcohol –OH stretch at 3620–3540 cm^−1^, and phenols –OH stretch at 3640–3530 cm^−1^ [59]. Previous researchers analyzed the FTIR spectra of the coffee husk biochar and they indicated that the cellulose structure of coffee husk disappeared during the carbonization process [61].

### 3.2 Electromagnetic shielding effectiveness (EM SE) analysis

**Fig. 7:**
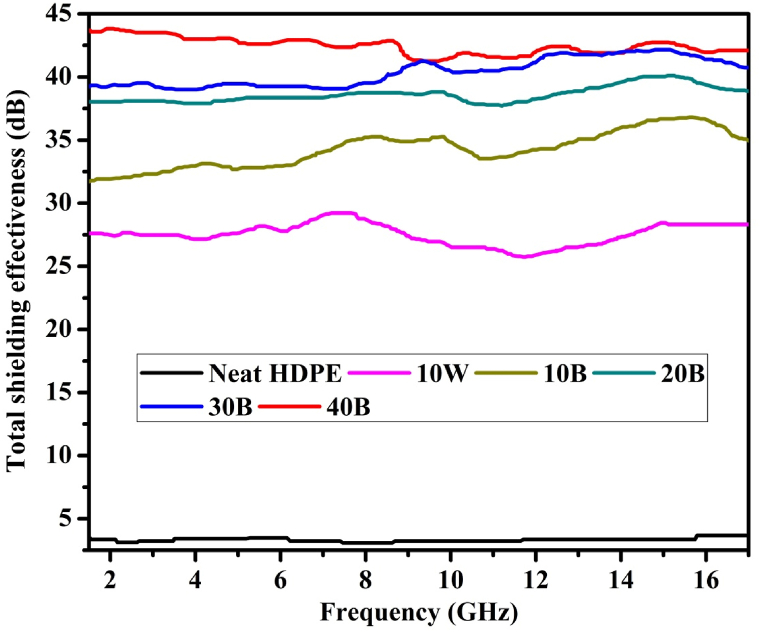
Total shielding effectiveness of pine wood biochar/HDPE composites.

During the EM SE analysis experiment, the transverse EM waves (waves perpendicular to the sample surface) were measured. The EM shielding tests were conducted by using ROHDE & SCHWARZ Vector network analyzer (ZVK-1127.8651) with a measuring capability of 10 MHz–40 GHz. As shown in Fig. 7, the total SE of 44.03 dB was recorded on the biochar/HDPE composite sample containing 40 % pine wood biochar. The minimum total SE of 0.5 dB was recorded on the neat HDPE sample. Generally, the total SE of the composite has significantly improved with increasing of the biochar loading. There were negligible variations of SE for each composites corresponding to the EM wave frequency increment. Neat HDPE shows the least EM SE compared with other composites. Xu, L. et al. [62], reported that the minimum SE of any EM shielding materials for industrial use should be between 10 and 30 dB and we improved it by 11.4 %. Therefore, the pine wood biochar/HDPE composite can be used for areas requiring SE of up to 40 dB.

The weight of EM shielding materials should be as low as possible. To determine the weight of the high EM shielding materials, the mass of biochar/HDPE composite samples having a uniform dimension of 80 mm × 12 mm x 3 mm were measured by using a digital balance. The density of pine wood biochar/HDPE-based composites showed the minimum comparable density compared with the density of different biochars from literature [63]. As shown in Table 6, the density of each sample was measured three times. The average density of net HDPE sample was 0.7603 g/cm^3^. The addition of 40 wt percentage of the pine wood biochar increased the density of the composite to the average density of 0.953 g/cm^3^. The increment of the density with the addition of 40 wt percentages of pine wood biochar is negligible (0.1927 g/cm^3^) with a supper EM wave SE.

**Table 6:** Mass and density measurement of biochar/HDPE samples.

Sample		Mass (g)	Density (g/cm^3^)	Average density (g/cm^3^)
**Neat HDPE**	1	2.115	0.734	0.7603 ± 0.026
	2	2.257	0.784	
	3	2.197	0.763	
**40B**	1	2.467	0.856	0.953 ± 0.097
	2	2.957	1.0267	
	3	2.814	0.977	

The addition of pine wood biochar, which acts as the conductive filler into the HDPE matrix increased the EM SE of the composites. The basic parameters that have been considered during the SE analysis in this study were the pine wood biochar loadings, the pyrolysis temperature, and the frequency of the EM wave. A high temperature pyrolysis of biomass can accelerates the conversion of SP^3^ C-X (X: C, O, H, etc.) bond into aromatic SP^2^ C=C bond resulting in the generation of graphitized carbon. The graphitized carbon, C=C bond links with each other and can forms a two-dimensional plane after the volatile matters has left the biomass during pyrolysis. This can creates the movement of numerous free electrons along the plane, which significantly increases the electrical conductivity that improves the EM SE of the composite. However, even though some volatile matters have been removed during the pyrolysis, some heteroatoms (N, O, P etc.) can be remained and creates different electronegativity between carbon atoms and heteroatoms. These heteroatoms will act as a polarization agent which induces dipole polarization and electronic polarization [64], and the improved polarization (interfacial and dipolar polarization) enables EM absorption.

**Fig. 8:**
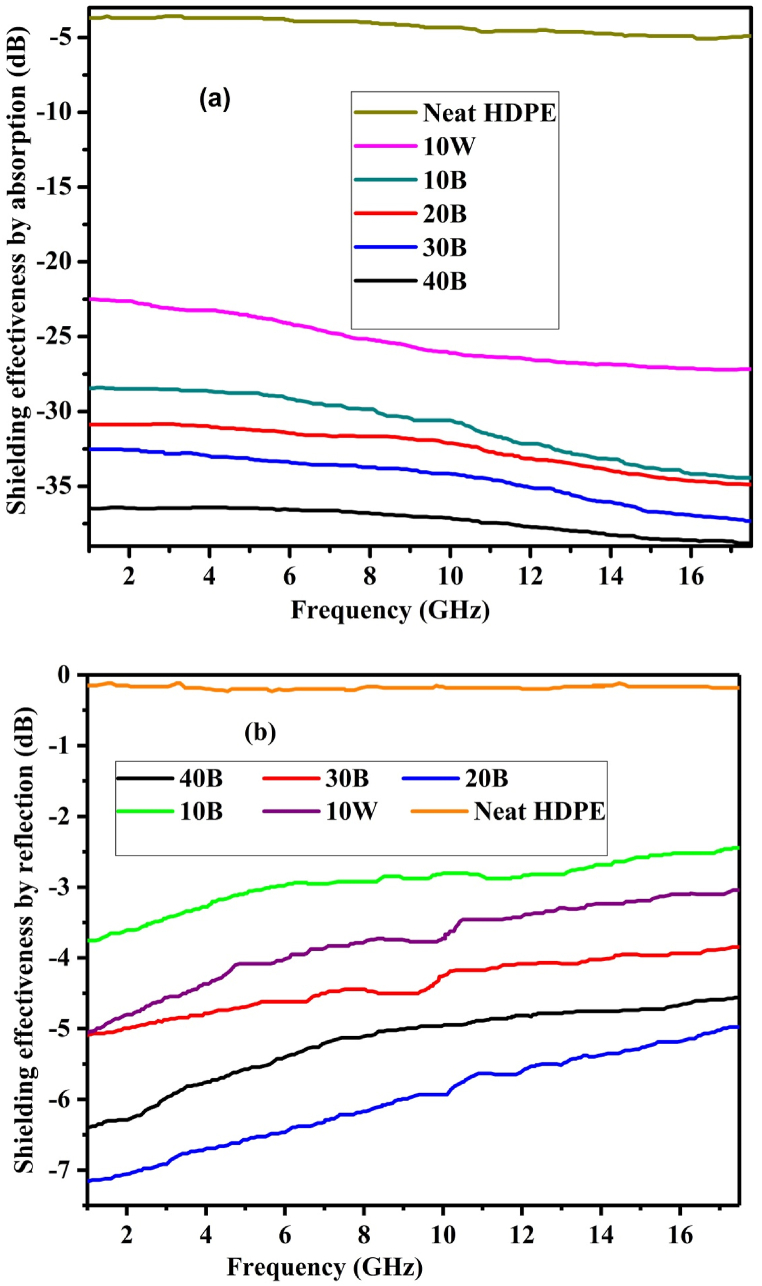
Shielding effectiveness of biochar/HDPE composite: a) absorption b) reflection.

This work clearly shows the advantages of using pyrolyzed biochar in comparison with non-pyrolyzed biomass for EM shielding application. The EM SE of a composite can be achieved due to the reflection of EM wave from the surface of the composite, and or the absorption of EM wave into the composites. Fig. 8 (a) shows the absorption SE of pine wood biochar/HDPE samples at different weight percentages of the biochar. The 40B sample showed the maximum absorption capacity in the frequency ranges of 1–18 GHz and the neat HDPE sample showed the minimum absorption capacity as shown in Fig. 8 (a). In a general trend, the absorption SE of the composite was increased with increasing of the biochar contents. The absorption SE of the composite was also increased with increasing of the frequency of the EM wave. However, the reflection shielding capacity of samples had been seen decreased as the frequency of the EM wave increased as shown in Fig. 8 (b). There were also variations of reflective SE of the composites when different weight percentages of biochar are added. The maximum reflection shielding was recorded on the 20B sample and the least reflection shielding was attained by the neat HDPE sample as shown in Fig. 8 (b). As depicted in Fig. 8, the EM reflective SE of 10W/HDPE composite was greater than the 10B/HDPE composite. However, for the absorption shielding applications the porous filler biochar/HDPE composites gave higher EM SE with increasing trends as the biochar loading is increased. The superior absorption EM SE of biochar/HDPE composites are related with the presence of the graphitized carbon with higher specific surface area, pore volume, and pore diameter as indicated in the BET analysis results. In this research, a very high (44.03 dB) SE was achieved by using the cost effective and environmental eco-friendly 40B biochar and recycled HDPE composite with a simple fabrication technique of melt blending and hot compression molding.

### 3.3 Thermal property analysis

#### 3.3.1 Thermogravimetric analysis (TGA)

**Fig. 9:**
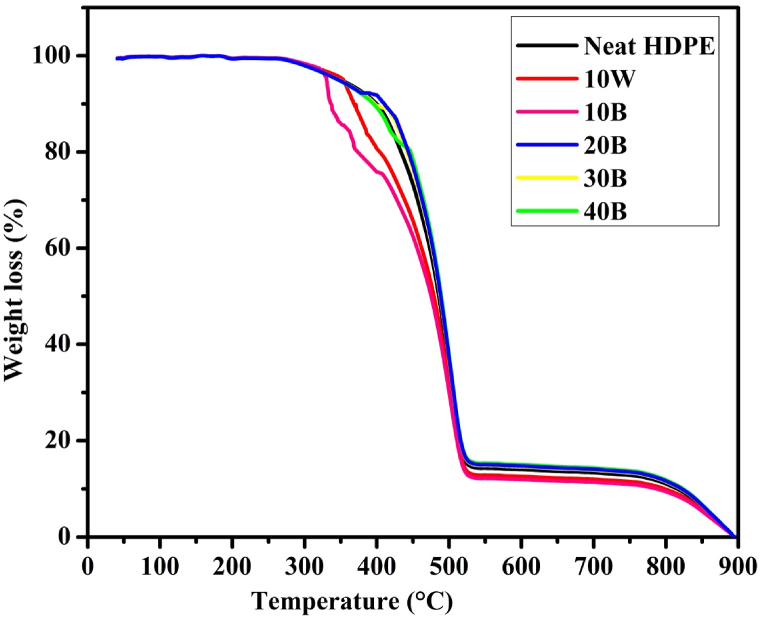
Thermogravimetric analysis of biochar/HDPE composites.

The TGA was done to determine the degradation and thermal stability of the recycled HDPE and pine wood biochar composites. We used it to measure the weight changes of the recycled HDPE/biochar composite as the rate of temperature is increased from 22 to 900 °C. The composites showed one-step degradation as shown in Fig. 9. The temperature at which the initial degradation of biochar/HDPE composites begin to degrade was observed to occur at around 350 °C. This initial degradation temperature determines the thermal stability of each composite. For neat HDPE and 10W samples, the initial degradation temperatures were seen to begin at around 320 °C and 345 °C respectively. The initial degradation temperatures of the composites have been seen increased as the filler biochar contents are increased. When the filler content (biochar) has been increased to 40B, the initial degradation temperature was increased to 400 °C as shown in Fig. 9. At the temperature of around 515 °C, most constituents of the biochar/HDPE composites degraded into ashes with a weight loss of around 90 %. The biochar/HDPE composites and the neat HDPE fabricated sample showed comparable thermal stability compared to the preliminary studies. The results showed that neat HDPE sample has lower thermal stability than biochar/HDPE composites with an onset degradation temperature of 320 °C, and it was fully degraded at around 515 °C.

#### 3.3.2 Differential thermal analysis (DTA)

**Fig. 10:**
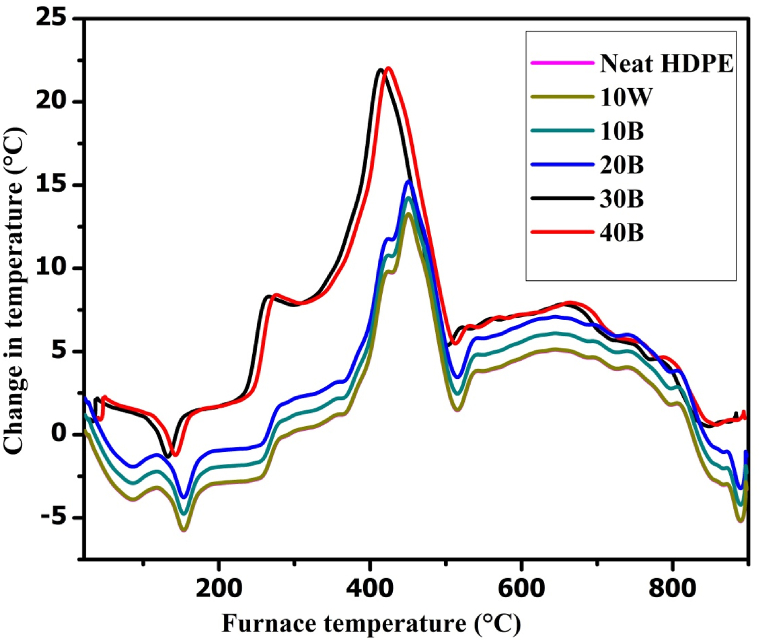
Differential thermal analysis of biochar/HDPE composites.

Differential thermal analysis (DTA) was done to determine the heat flow stability and the crystallization of the biochar/HDPE composites in comparison with the temperature of the reference material by heating at a predetermined uniform rate of 20 ^°^C/min from the gas temperature (0 °C–1500 °C) to 900 °C. Thermal stability is a limiting factor for composites integrated with polymeric materials. DTA measures the change in temperature associated with physical or chemical changes during the gradual heating of the biochar/HDPE composites and plots the change in temperature. In this experiment, the effects of the formation of crystalline structures, decomposition reactions, oxidation and reduction reactions, and other chemical reactions on the thermal stability of biochar/HDPE composites were studied by using DTA. As shown in Fig. 10, small exothermic and endothermic reactions in the composites occurred at a furnace temperature of 135 °C leading to the decomposition of low temperature organic matters and the dehydration of moistures respectively. Furthermore, Fig. 10 indicated that the highest rate of exothermic reaction occurred at 417.6 °C for a 30B and 40B biochar/HDPE composites with a change in temperature of around 21 °C which indicates the formations of a high crystalline biochar with a full decomposition of organic matters. Furthermore, an endothermic reaction occurred on all biochar/HDPE composites at a furnace temperature of 515 °C due to the degradation of HDPE constituent and other high temperature volatile matters.

#### 3.3.3 Thermal conductivity analysis

The thermal conductivity of the composites of neat HDPE, 10W, 10B, 20B, 30B, and 40B were determined in the temperature ranges of 24–60 °C by using thermal conductivity measuring device (G.U.N.T WL 374). The sizes of the samples were taken to be the same for all tests. During the experimental measurements, k = $\frac{{\dot{Q}}_{w}h}{A\Delta T}$ as a thermal conductivity, ${\dot{Q}}_{w}$ as a heat flow rate, h as a thickness, A as area of samples, and ΔT as the change of temperature = (T_1_-T_2_) were considered as shown in Table 7. We used a thickness of 3 mm and area of 1256 mm^2^ for each type of sample.

**Table 7:** Thermal conductivity measurement of the biochar/HDPE composite samples.

**Sample**	**Heater (V)**	**Heater(A)**	${\dot{Q}}_{H}$**(W)**	${\dot{m}}_{w}$**(g/s)**	**T1w**	**T2w**	${\dot{Q}}_{w}$	**T**_**1**_	**T**_**2**_	**K (W/m K)**
**HDPE**	81 ± 1	0.45 ± 1	36.416 ± 3.616	1.4	16.93 ± 0.06	17.7 ± 0.2	4.48 ± 0.97	43.1 ± 1.5	21.36 ± 2.06	0.492 ±0.039
**10W**	79.66 ± 2.6	0.54 ± 0.04	43.19 ± 1.69	1.4	16.83 ± 0.33	17.73 ± 3.3	5.26 ± 0.5	46.53 ± 1.8	28.43 ± 1.5	0.695 ± 0.02
**10B**	81.3 ± 3.3	0.51 ± 0.09	41.7 ± 8.9	1.4	16.86 ± 0.16	19.2 ± 0.1	12.87 ± 0.87	56.16 ± 0.13	27.63 ± 2.23	1.144 ± 0.038
**20B**	86.3 ± 2.3	0.57 ± 0.04	49.4 ± 3.3	1.4	16.96 ± 0.26	19.56 ± 0.26	15.216 ± 0.006	50.96 ± 1.06	30.86 ± 1.06	1.809 ± 0.03
**30B**	90.6 ± 2.6	0.613 ± .043	55.68 ± 5.5	1.4	16.6 ± 0.26	19.96 ± 0.16	19.3 ± 1.16	51.36 ± 2.8	30.83 ± 2.3	2.219 ± 0.19
**40B**	90.3 ± 1.3	0.54 ± 0.06	49.41 ± 4.58	1.4	16.73 ± 0.23	20.36 ± 0.56	21.26 ± 1.95	55.3 ± 2.4	26.16 ± 0.76	1.749 ± 0.105

**Fig. 11:**
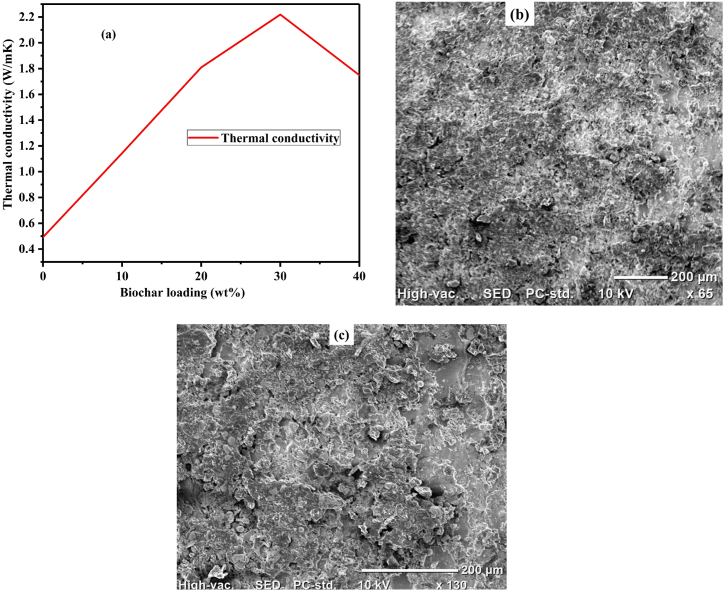
Thermal conductivity of biochar/HDPE composites: a) graph of conductivity b) SEM of 30B/HDPE composite, and c) SEM of 40B/HDPE composite.

**Fig. 12:**
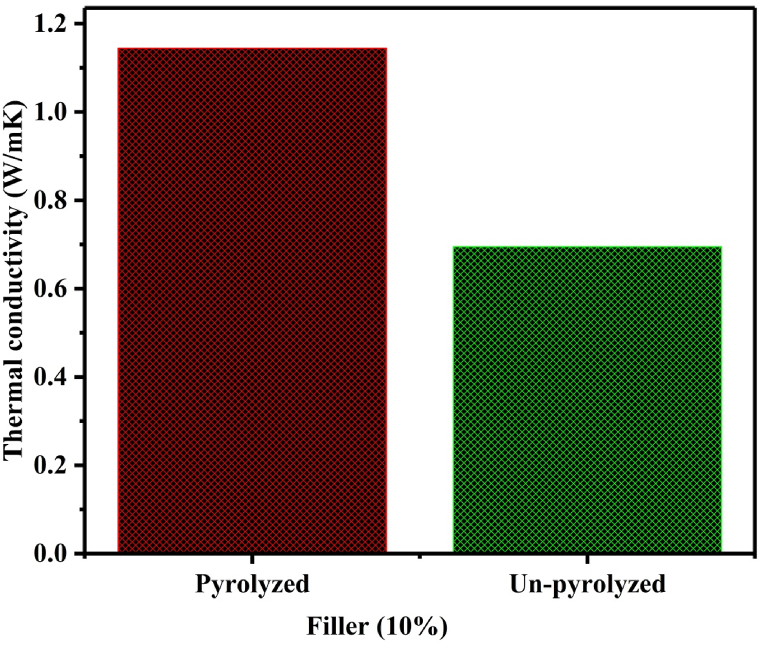
Effect of pyrolysis on thermal conductivity of biochar/HDPE composites.

In this experiment, the density and specific heat of water were taken as 1000 kg/m^3^ and 4180 J/kg.K respectively. The mass flow rate of water was measured by drop watch, and $\dot{Q}$_H_ as voltage x heater current, $\dot{Q}$_w_ as $\dot{m}$_w_ x C_p_ (T_1w_–T_2w_), $\dot{m}$_w_ as the mass flow rate of water, C_p_ as specific heat of water which is 4180 J/kg.K, and T_1w_ & T_2w_ as temperatures of water entering and leaving the system were considered. It is required to have thermally conductive EM shielding materials to safely dissipate the heat energy developed in the materials through EM wave absorption. The thermal conductivity of the composite at 30B was observed to be higher than the other biochar loadings as shown in Fig. 11 (a). Further increment of the biochar content to 40B decreased the thermal conductivity of biochar/HDPE composites. The thermal conductivity of 40B sample had been seen decreased due to the agglomeration effects and the formations of very high porosity as shown in Fig. 11 (b and c) leading to weak interconnection between filler and matrix. Furthermore, the effect of pyrolyzing on the thermal conductivity had been illustrated in Fig. 12. The pyrolysis process has effectively increased the thermal conductivity by 39.25 % for 10 % biomass filler used.

### 3.4 Electrical property analysis of biochar/HDPE composites

**Fig. 13:**
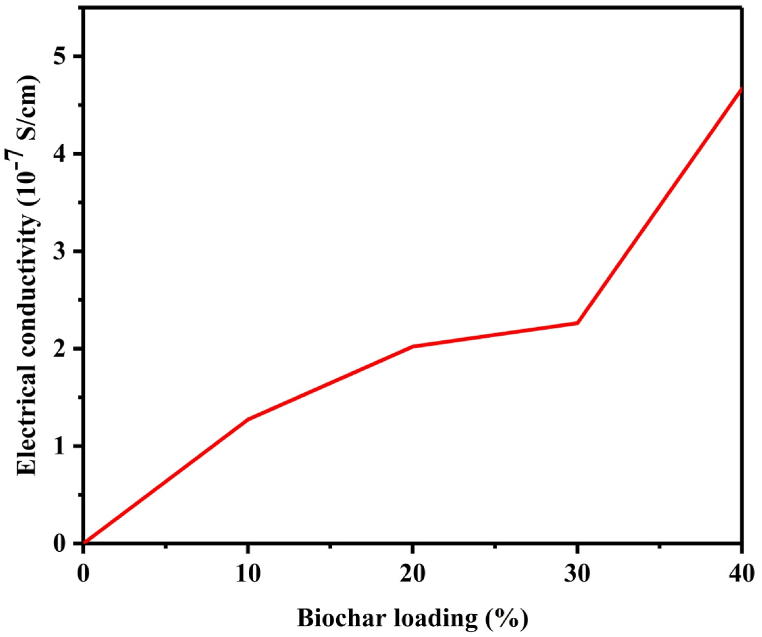
Electrical conductivity measurement and analysis results of biochar/HDPE composites.

Pine wood biochar and its composites with recycled HDPE were tested for their electrical resistivity and conductivity using a digital multi-meter (fluke 87 RMS true plus multi-meter). After measuring the resistivity and conductivity, the values were normalized with respect to the area of electrode contact and the distance between the electrodes [133] as shown in Table 8. The resistivity of neat HDPE was in the ranges of insulating materials which was measured to be between 10^15^ to 10^18^ Ω as shown in Table 8. However, when 10 % pyrolyzed pine wood biochar is added to HDPE, the resistivity of the composite was reduced by 54.27 % from the 10 % pine wood powder/HDPE composite. Thus, the incorporation of pine wood biochar showed a significant potential to improve the electrical properties of the resulting composites. Studies reported indicated that biochars containing high amount of fixed carbon had electrical conductivity between the ranges of 2300–3300 S/m [50,65]. The biochar/HDPE composites fabricated by melt blending combined with hot compression molding showed increased electrical conductivity with respect to increasing of biochar loading as shown in Fig. 13. This demonstrated that the conducting networks were already well established in the biochar/HDPE composites.

**Table 8:** Electrical resistance and conductivity of biochar/HDPE composites.

Sample	Measured Resistance (MΩ)	Normalized resistivity (MΩcm)	Normalized conductivity (S/cm)
HDPE	10^12^±0.0	8 × 10^12^±0.0	(8 ± 0)x 10^−18^
10W	11.103 ± 0.75	13.878 ± 0.93	(7.21 ± 0.93)x 10^−8^
10B	6.284 ± 0.244	7.855 ± 0.305	(1.273 ± 0.305)x 10^−7^
20B	3.959 ± 0.062	4.948 ± 0.0775	(2.021 ± 0.077)x 10^−7^
30B	3.537 ± 0.192	4.42125 ± 0.24	(2.262 ± 0.24)x 10^−7^
40B	1.713 ± 0.165	2.14125 ± 0.206	(4.67 ± 0.206)x 10^−7^

### 3.5 SEM analysis of biochar/HDPE composites

**Fig. 14:**
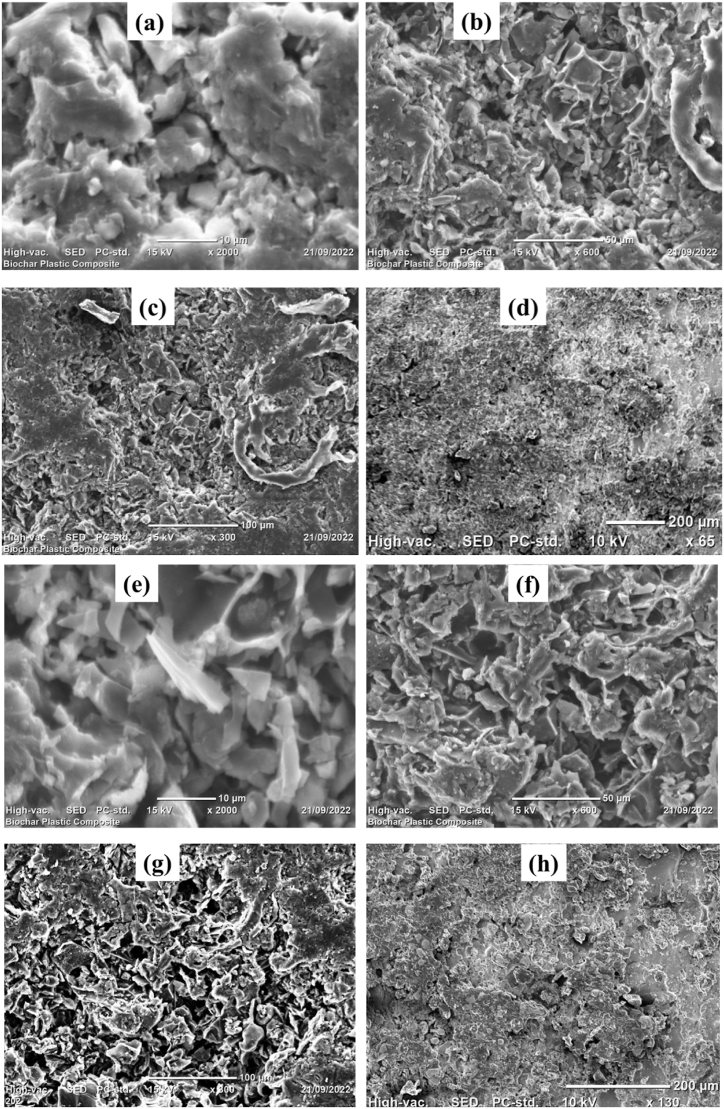
SEM results of 30B samples at a) 10 μm, b)50 μm, c) 100 μm, d) 200 μm, and 40B samples at e) 10 μm, f) 50 μm, g) 100 μm, and h) 200 μm.

The surface morphology of biochar/HDPE composites were studied by using SEM (JCM-6000Plus) operated at a voltage of 15 KV in the microstructural ranges of 200–10 μm. The SEM results of the biochar/HDPE composites are shown in Fig. 14 (a, b, c, d, e, f, g, and h). As shown in Fig. 14 (b and e), the thermal properties, electrical properties, and density of 30B and 40B biochar/HDPE composites showed the direct relations to their morphological arrangements and crystallinity. It is very clear to examine the high crystallinity of the 40B biochar/HDPE composite as shown in Fig. 14 (e). Furthermore, the SEM analysis of 30B and 40B at 100 μm indicated that the conductivity depends on the existence of high intensity of crystallinity.

## 4 Conclusion

Electromagnetic waves emitted from the natural and electronic sources are becoming a challenge for electronics, human health, and the environment. The design and development of materials with the required capacity to shield electromagnetic energy from the transmission can be evaluated by the conductivity, constituent functional groups, thermal stability, microstructural morphology, and polarization of the shielding surface of the materials**.** The agricultural residues and agro-industry wastes residues’ biochars are resourcefully available materials with the required electromagnetic shielding capacity synthesized by slow pyrolysis. The solid substrates from biochar/HDPE composites of the shielding materials can be fabricated by using low-cost melt-blending combined with hot-compression molding with the required conducting networks established in the biochar/HDPE composites. In conclusion, the shielding effectiveness of the biochar/HDPE composites was increased in proportion to the highest thermal conductivity, electrical conductivity, thermal stability, and high specific surface area of the biochar. From this research work, the following results are obtained:

(a) The carbon loading effects were controlled by the biomass source types, pyrolysis conditions, and biochar weight percentages
(b) From the experimental investigation, around 11.4 % of higher shielding effectiveness was achieved compared to the previous research results.
(c) The biochar/HDPE composite with 30 wt% PW biochar showed a good electromagnetic shielding effectiveness, thermal, and electrical properties.
(d) The 40 wt% PW biochar/HDPE composite showed the highest electrical conductivity of 4.67 × 10^−7^ S/cm.

## Funding

No funding for this work.

## Data availability

No data availability.

## CRediT authorship contribution statement

**Amanu Asmare Fenta:** Writing - original draft, Methodology, Formal analysis, Data curation. **Addisu Negash Ali:** Writing - review & editing, Validation, Supervision, Methodology, Data curation, Conceptualization.

## Declaration of competing interest

The authors declare that they have no known competing financial interests or personal relationships that could have appeared to influence the work reported in this paper.

## References

[1] Kruželák J. (2021). Progress in polymers and polymer composites used as efficient materials for EMI shielding. Nanoscale Adv..

[2] Yang F. (2019). Microwave-absorbing properties of room-temperature ionic liquids. J. Phys. Appl. Phys..

[3] Dittrich B. (2014). Flame-retardancy properties of intumescent ammonium poly (phosphate) and mineral filler magnesium hydroxide in combination with graphene. Polymers.

[4] Raagulan K., Kim B.M., Chai K.Y. (2020). Recent advancement of electromagnetic interference (EMI) shielding of two dimensional (2D) MXene and graphene aerogel composites. Nanomaterials.

[5] Halgamuge M.N., Abeyrathne C.D., Mendis P. (2010). Measurement and analysis of electromagnetic fields from trams, trains and hybrid cars. Radiat. Protect. Dosim..

[6] Saini P. (2019).

[7] Ayub S. (2021). Preparation methods for graphene metal and polymer based composites for EMI shielding materials: state of the art review of the conventional and machine learning methods. Metals.

[8] Von Klemperer C.J., Maharaj D. (2009). Composite electromagnetic interference shielding materials for aerospace applications. Compos. Struct..

[9] Pickering K.L. (2007). Optimising industrial hemp fibre for composites. Compos. Appl. Sci. Manuf..

[10] Wanasinghe D., Aslani F. (2019). A review on recent advancement of electromagnetic interference shielding novel metallic materials and processes. Compos. B Eng..

[11] Chung D. (2001). Electromagnetic interference shielding effectiveness of carbon materials. Carbon.

[12] Tolvanen J. (2019). Biodegradable multiphase poly (lactic acid)/biochar/graphite composites for electromagnetic interference shielding. Compos. Sci. Technol..

[13] Zhan Z. (2019). Ultrastrong and conductive MXene/cellulose nanofiber films enhanced by hierarchical nano-architecture and interfacial interaction for flexible electromagnetic interference shielding. J. Mater. Chem. C.

[14] Dai S.-W. (2021). Bamboo-inspired mechanically flexible and electrically conductive polydimethylsiloxane foam materials with designed hierarchical pore structures for ultra-sensitive and reliable piezoresistive pressure sensor. Compos. B Eng..

[15] Wu J. (2016). Effect of electrophoretic condition on the electromagnetic interference shielding performance of reduced graphene oxide-carbon fiber/epoxy resin composites. Compos. B Eng..

[16] Sharif F. (2017). Segregated hybrid poly (methyl methacrylate)/graphene/magnetite nanocomposites for electromagnetic interference shielding. ACS Appl. Mater. Interfaces.

[17] Santhosi B., Ramji K., Rao N.M. (2020). Design and development of polymeric nanocomposite reinforced with graphene for effective EMI shielding in X-band. Phys. B Condens. Matter.

[18] Karuppasamy K. (2016). Ionic liquid incorporated nanocomposite polymer electrolytes for rechargeable lithium ion battery: a way to achieve improved electrochemical and interfacial properties. J. Ind. Eng. Chem..

[19] Kwiatkowski M., Kalderis D. (2020). A complementary analysis of the porous structure of biochars obtained from biomass. Carbon Letters.

[20] Wang L. (2020). Green immobilization of toxic metals using alkaline enhanced rice husk biochar: effects of pyrolysis temperature and KOH concentration. Sci. Total Environ..

[21] Dai Y. (2020). Combined effects of biochar properties and soil conditions on plant growth: a meta-analysis. Sci. Total Environ..

[22] Suliman W. (2016). Influence of feedstock source and pyrolysis temperature on biochar bulk and surface properties. Biomass Bioenergy.

[23] Arrigo R., Bartoli M., Malucelli G. (2020). Poly (lactic acid)–biochar biocomposites: effect of processing and filler content on rheological, thermal, and mechanical properties. Polymers.

[24] Afroza Khatun M. (2019). Physical, mechanical, thermal and morphological analysis of date palm mat (DPM) and palmyra palm fruit (PPF) fiber reinforced high density polyethylene hybrid composites. Advanced Materials Science.

[25] Sameer A., Khalaf E. (2019). An investigation of the improvements of mechanical and thermal properties of high-density polyethene/nano clay composites. European Mechanical Science.

[26] Oliveira L.S. (2008). Coffee oil as a potential feedstock for biodiesel production. Bioresour. Technol..

[27] Balemlay A.M. (2021).

[28] Habte L., Mulatu D., Ahn J.W. (2018). Applications of Sugarcane by-products to mitigate climate change in Ethiopia. J. Energy Eng..

[29] Awad S.A., Khalaf E.M. (2020). Characterization and modifications of low-density poly ethylene-nano cellulose crystalline composites. Suranaree Journal of Science & Technology.

[30] Wright M. (2010). Techno-economic comparison of biomass-to-biofuels pathways. Fuel.

[31] Yang H. (2007). Characteristics of hemicellulose, cellulose and lignin pyrolysis. Fuel.

[32] Demirbas A., Arin G. (2002). An overview of biomass pyrolysis. Energy Sources.

[33] Ahmad, M., RajapakshaA. U., Lim JE, Zhang M., Bolan DM, Vithanage M., Lee SS, Ok SY, 2014: p. 19-33. 10.1016/j.chemosphere.2013.10.071 24289982

[34] Kang B.-S. (2006). Fast pyrolysis of radiata pine in a bench scale plant with a fluidized bed: influence of a char separation system and reaction conditions on the production of bio-oil. J. Anal. Appl. Pyrol..

[35] Pattanakul C. (1991). Properties of recycled high density polyethylene from milk bottles. J. Appl. Polym. Sci..

[36] Santos I.d.S.d. (2020). Physical and thermal properties of eucalyptus wood charcoal. Cerne.

[37] Zhao Y. (2014). Study on the water-heat coupled phenomena in thawing frozen soil around a buried oil pipeline. Appl. Therm. Eng..

[38] Hankalin V., Ahonen T., Raiko R. (2009).

[39] Tushir S. (2021). Physical and thermal properties of corn cob powder blended mud cup (khulad). Indian J. Agric. Sci..

[40] Aminudin E. (2017). MATEC Web of Conferences.

[41] Sonawane A., Pathak S.S., Pradhan R.C. (2020). Physical, thermal, and mechanical properties of bael fruit. J. Food Process. Eng..

[42] Wang Y. (2010). Convective heat transfer of the Bunsen flame in the UL94 vertical burning test for polymers. J. Fire Sci..

[43] Zhu Q. (2014).

[44] Enders A., Lehmann J. (2012). Comparison of wet-digestion and dry-ashing methods for total elemental analysis of biochar. Commun. Soil Sci. Plant Anal..

[45] Zhang Q. (2017). The dynamic mechanical analysis of highly filled rice husk biochar/high-density polyethylene composites. Polymers.

[46] Gedler G. (2016). Enhanced electromagnetic interference shielding effectiveness of polycarbonate/graphene nanocomposites foamed via 1-step supercritical carbon dioxide process. Mater. Des..

[47] Menberu Z.S. (2021).

[48] Nicholas K., Julius S. (2019). Pryloysis of coffee husks for biochar production. J. Environ. Protect..

[49] Okabe T., Saito K. (1994). Ecomaterials.

[50] Kane S. (2021). Physical and chemical mechanisms that influence the electrical conductivity of lignin-derived biochar. Carbon Trends.

[51] Zhang Y. (2022).

[52] Santos R.V. (2022). Assessment of biomass and biochar of maritime pine as a porous medium for water retention in soils. Energies.

[53] Bayartsengel B. (2021). 5th International Conference on Chemical Investigation and Utilization of Natural Resource (ICCIUNR-2021).

[54] Kamali M. (2022). Acclimatized activated sludge for enhanced phenolic wastewater treatment using pinewood biochar. Chem. Eng. J..

[55] Purevsuren B. (2018). Pyrolysis of pine wood and characterisation of solid and liquid products. Mongolian Journal of Chemistry.

[56] Kane S. (2022). Reducing the environmental impacts of plastics while increasing strength: biochar fillers in biodegradable, recycled, and fossil-fuel derived plastics. Composites Part C: Open Access.

[57] Michczyńska D.J. (2018). Different pretreatment methods for 14C dating of Younger Dryas and Allerød pine wood (Pinus sylvestris L.). Quat. Geochronol..

[58] Kane S., Ryan C. (2022). Biochar from food waste as a sustainable replacement for carbon black in upcycled or compostable composites. Composites Part C: Open Access.

[59] Nandiyanto A.B.D., Oktiani R., Ragadhita R. (2019). How to read and interpret FTIR spectroscope of organic material. Indonesian Journal of Science and Technology.

[60] Bajwa D.S. (2019). Characterization of bio-carbon and ligno-cellulosic fiber reinforced bio-composites with compatibilizer. Construct. Build. Mater..

[61] Veiga T.R.L.A. (2017). Different plant biomass characterizations for biochar production. Cerne.

[62] Xu L. (2018). Efficient electromagnetic interference shielding of lightweight carbon nanotube/polyethylene composites via compression molding plus salt-leaching. RSC Adv..

[63] Natalio F. (2020). Sustainable lightweight biochar-based composites with electromagnetic shielding properties. ACS Omega.

[64] Zhao H. (2019). Biomass-derived porous carbon-based nanostructures for microwave absorption. Nano-Micro Lett..

[65] Nan N. (2016). The effect of bio-carbon addition on the electrical, mechanical, and thermal properties of polyvinyl alcohol/biochar composites. J. Compos. Mater..

